# Metagenomic profiling of placental tissue suggests DNA virus infection of the placenta is rare

**DOI:** 10.1099/jgv.0.001677

**Published:** 2021-11-01

**Authors:** Adam A. Witney, Sean Aller, Blair L. Strang

**Affiliations:** ^1^​ Institute for Infection and Immunity, St George’s, University of London, London SW17 0RE, UK

**Keywords:** herpes, metagenomic profiling, placenta, virus

## Abstract

It is widely recognized that pathogens can be transmitted across the placenta from mother to foetus. Recent re-evaluation of metagenomic studies indicates that the placenta has no unique microbiome of commensal bacteria. However, viral transmission across the placenta, including transmission of DNA viruses such as the human herpesviruses, is possible. A fuller understanding of which DNA virus sequence can be found in the placenta is required. We employed a metagenomic analysis to identify viral DNA sequences in placental metagenomes from full-term births (20 births), pre-term births (13 births), births from pregnancies associated with antenatal infections (12 births) or pre-term births with antenatal infections (three births). Our analysis found only a small number of DNA sequences corresponding to the genomes of human herpesviruses in four of the 48 metagenomes analysed. Therefore, our data suggest that DNA virus infection of the placenta is rare and support the concept that the placenta is largely free of pathogen infection.

## Introduction

Vertical transmission of pathogens from mother to child is a mechanism of pathogen dissemination within human populations [1]. This can occur by several well-characterized mechanisms, including post-partum infection at birth and via breast milk [1]. However, vertical transmission of pathogens from mother to foetus across the placenta *in utero* is very poorly understood, and conflicting data regarding bacterial colonization of the placenta have led to recent re-evaluation of the placenta as a sterile environment.

The womb is thought of as an environment free of bacteria as it is well documented that germ-free neonates can be delivered by caesarean section [2, 3], an observation that can be recapitulated in animal models [3]. However, there have been reports of bacteria found in placental tissue [4–12], amniotic fluid [7] and the foetus [13], leading to reports that the placenta harbours a unique microbiome of non-pathogenic commensal bacteria. Arguably the most prominent reports of a unique placental microbiome have been produced by Aagaard and colleagues [4–6], who reported the detection of a wide range of commensal bacterial DNA sequences in placental tissue using methods such as bacterial 16S sequencing and metagenomic DNA profiling.

There have been several challenges to the aforementioned reports. Notably, these studies reported that only very low levels of bacteria were present and did not demonstrate that viable bacteria were detected [14]. It has been further suggested that the reported placental microbiome could be bacteria found in maternal blood within the placental tissue analysed or come from another maternal site [14]. However, there is unlikely to be sufficient maternal blood within placental tissue to justify this reasoning [15].

The strongest rebuttal to reports of a placenta microbiome has been offered by data that detection of bacterial DNA within placental tissue was probably due to contamination of samples during preparation of genomic DNA via the DNA purification kits used, or via contamination with bacteria found in the environment during sample collection and/or DNA purification [16–20]. Similarly, the presence of bacteria in amniotic fluid [7] has been challenged by work reporting that the bacterial sequence signals found in amniotic fluid samples were indistinguishable from background controls [21]. Furthermore, a recent survey of the placentas from many hundreds of patients has indicated that the placenta has no obvious unique microbiome and that nearly all detection of bacteria in the survey was connected to contamination of reagents with bacterial DNA and acquisition of bacteria during labour and/or delivery [22].

The recent re-evaluation of what bacteria are found in placental tissue stimulated us to evaluate what viruses can be found in the placenta. It is known that viruses can pass through the placenta, be transmitted to the foetus and cause disease [1]. Some of the most prominent viruses vertically transmitted across the placenta are those with DNA genomes, such as the human herpesvirus (HHV), herpes simplex virus (HSV), human cytomegalovirus (HCMV) and varicella-zoster virus (VZV) [1]. However, a fuller understanding of the prevalence of herpesvirus infection of the placenta and how these viruses replicate and cause disease in the placenta or affect pregnancy outcomes is required [1, 23, 24]. Plus, there have been contrasting reports of which herpesviruses can be found in placental tissue. It has been reported that HCMV and HSV can be found in full-term placental and decidual tissue in the presence and absence of several common sexually transmitted pathogenic non-commensal bacteria (e.g. chlamydia) [25]. It is unknown what effect these bacterial infections could have on herpesvirus replication and pathogenesis. Moreover, it has been observed that more placental tissues contain HCMV and a pathogenic bacterial species than HCMV alone [25], suggesting pathogenic bacterial infection promotes HCMV infection. However, more recent studies have reported that DNA from HSV-1, HSV-2 or HCMV could not be found in placental DNA from full-term pregnancies, pre-term pregnancies, pregnancies with pre-eclampsia or pregnancies with foetal growth restriction [26].

Also, other herpesviruses can utilize the placenta for vertical transmission, which leads to disease. For example, it has been demonstrated that HHV-6A, -7 and -8 can replicate in cells derived from placental tissue *in vitro* and HHV-6A, -6B and -7 could be involved in disease during pregnancy [26–33]. Specifically, a recent report has demonstrated that the only viral RNA that could be reliably detected in cases of pre-eclampsia was from either HHV-6A or HHV-6B genomes and there was a strong association between the presence of chromosomally inherited HHV-6A or -6B and pre-eclampsia [26]. However, it is unknown if HHV-6 viruses have an obvious impact on other pathologies during pregnancy or if there is any relationship between HHV-6 infection and bacterial infection during pregnancy.

There has also been contrasting data presented on which DNA viruses other than herpesviruses may be found in the placenta. There are reports that adenovirus [34], adeno-associated virus [35], papillomavirus [36] and polyomavirus [37] can infect human or murine placental cells *in vitro*, suggesting that viruses from these families may be found in the placenta. However, a recent study has indicated that adenovirus and papillomavirus DNA could not be found in placental DNA extracts from full-term pregnancies, pre-term pregnancies, pregnancies with pre-eclampsia or pregnancies with foetal growth restriction [26]. Plus, it is as yet unknown if there are the links between placental infection by these viruses and disease in humans.

Therefore, it was possible that any of the aforementioned DNA viruses could be found in the placenta, alone or in combination, and that non-commensal bacterial infection could have a role in promoting virus infection. To understand which viruses with DNA genomes could be found in the placenta we performed an analysis of a published metagenomic dataset of genomic DNA isolated from the placental tissue of several patient groups, including those from preterm births and/or antenatal infections.

## Methods

### Microbiome data from public repositories

Microbiome-derived DNA sequences from a range of body sites were selected (filtered by file type ‘FASTQ’ and data ‘WGS’) and downloaded from either the National Institutes of Health (NIH) Human Microbiome Project (HMP) database or the NIH Integrative Human Microbiome Project (iHMP) database [38–40]. Accession numbers of sequences analysed can be found in Table S1 (available in the online version of this article). In addition, 52 metagenomes sequenced from samples from the upper reproductive tract were downloaded [41]. Upper reproductive tract sequences are available from the EBI ENA database with project accession PRJEB24147 [41].

### Analysis of DNA sequencing using metagenomic profiling tools

Metagenomic sequence reads were trimmed using Trimmomatic (version 0.39) [42] in paired-end mode to yield sets of high-quality paired reads. Trimmed reads were filtered to remove any human sequences using Kraken2 [43] against a human genome sequence-specific database. Taxonomic classifications were made against several databases using Kraken2; the databases included the full standard database and a viral genome-specific database. The output of Kraken2 consists of the number of unique reads across multiple taxonomies. For the purposes of the present study, only reads categorized under Viruses by Kraken2 were analysed. Normalized read counts were generated by dividing by the sample read count and scaling to 10^6^.

Where indicated in the text, reads were also analysed using Kraken2 by comparing reads to a database of human herpesvirus genomes created by downloading all human herpesvirus genomes from NCBI Viral Neighbor assemblies. A total of 1379 genomes were analysed, comprising 70 HSV-1 genomes (5 % of the genomes analysed), 64 HSV-2 genomes (4.6 %), 202 VZV genomes (14.6 %), 659 EBV genomes (47.8 %), 333 HCMV genomes (24.1 %), seven HHV-6A genomes (0.5 %), nine HHV-6B genomes (0.65 %), three HHV-7 genomes (0.2 %) and 32 KSHV genomes (2.3 %).

Raw untrimmed reads were also converted to bam format using Picard, and classified using the GATK PathSeq pipeline using default settings [44]. Analysis and plotting were performed using R (v4.0.4) with the tidyverse package [45].

### Placental metagenome data

**Fig. 1.**
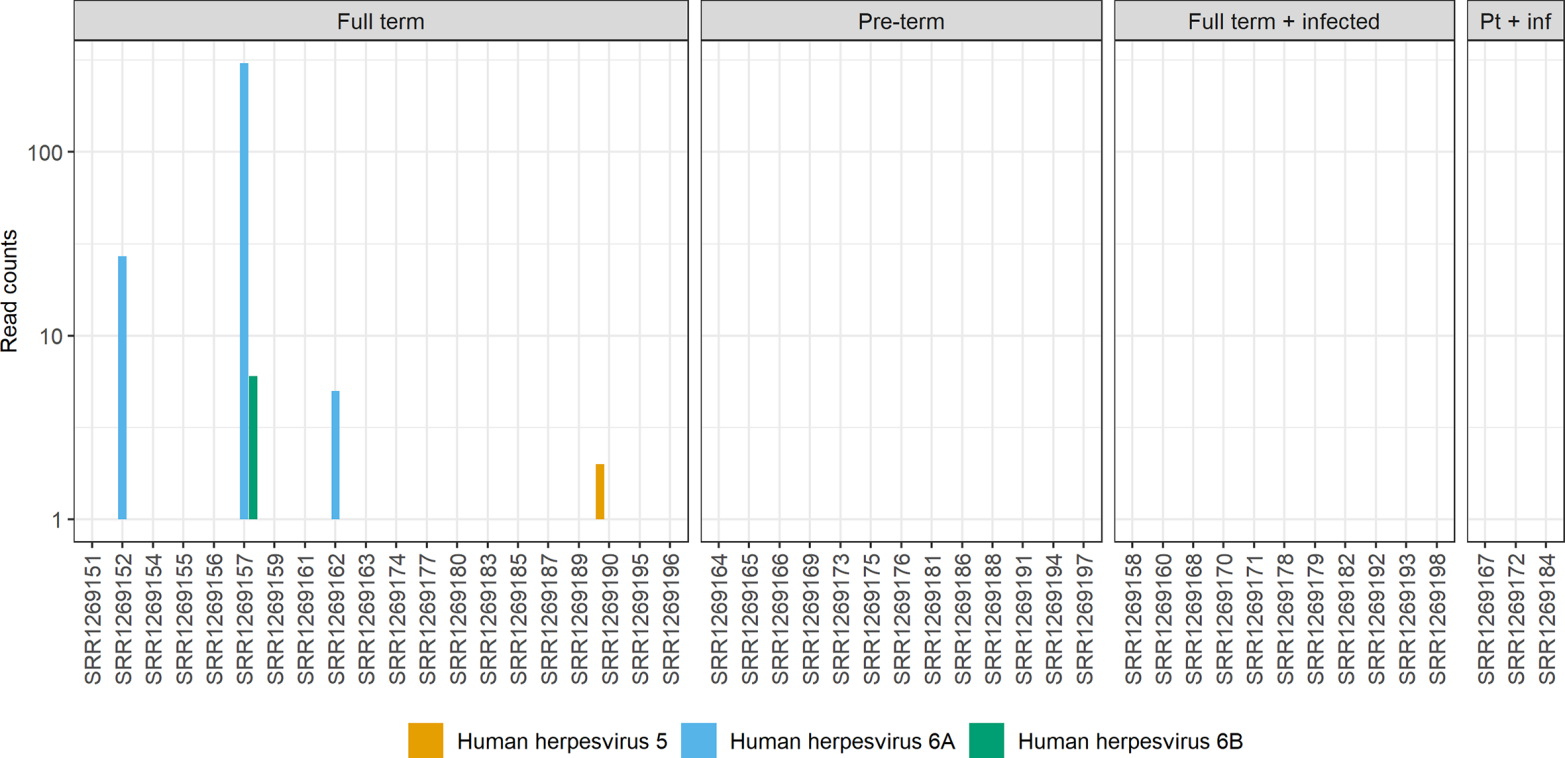
Number of reads corresponding to viral genomes in placental metagenomes. The sample accession of each metagenomic sample is noted on the *x*-axis. Metagenomic samples came from either full-term pregnancies, pre-term pregnancies, full-term pregnancies that had reported antenatal infection or pre-term pregnancies that had reported antenatal infections (Pt + inf). Accession SRR1269153 was removed as it had a higher than expected level of phiX174-derived reads, which were probably misclassified as other bacterial phage viruses (data not shown). HCMV is shown as human herpesvirus 5.

Forty-eight placental metagenomic sequences [5] were very kindly provided by Kjersti Aagaard and colleagues via the NCBI database of Genotype and Phenotype (request no. #65088-2; see the Acknowledgments for further information). The interactions with patients, preparation of samples and DNA sequencing methodology used to generate this metagenomic data have been previously described in detail [5]. Briefly, placental samples were collected immediately after delivery. Several tissue sections (between four and six) were taken from the same area of each placenta (with the maternal decidua and foetal chorion-amnion tissues removed), potentially gathering tissue from the placental arteries, placental vein and villous. DNA was extracted from the combined four to six sections of each placenta using a MO-BIO PowerSoil DNA Isolation Kit (MO-BIO Laboratories), which should extract all DNA from the tissue, including human genomic DNA, viral genomic DNA, bacterial genomic DNA and phage genomic DNA. All DNA was sequenced using the Illumina HiSeq 2500 platform. The sequencing data from the combined sections of each placenta were assigned an accession number used in Figs 1, S1 and S2.

Where indicated in the text, the estimated genome coverage of sequence reads was calculated as: ((no. of reads×read length×2)/genome length).

## Results

### Investigation of viral DNA sequence reads in placental metagenomic DNA

The largest public database of placental metagenomes available was generated from the aforementioned study by Aagaard *et al.* [5], which contained metagenomic data from different patient groups; those pregnancies that had delivered at full term (20 births), pregnancies that had delivered spontaneously preterm (<37 weeks) (13 births), full-term pregnancies that had reported infections [antepartum sexually transmitted infections (e.g. gonorrhoea, chlamydia or syphilis), urinary tract infections or systemic infections (e.g. pneumonia)] (12 births) and pregnancies that were both preterm and had reported infections (three births). Information on sample collection and preparation can be found in the placental metagenome data portion of the Materials and Methods section.

Based on previously reported factors such as accuracy and ease of use [46], a workflow based on Kraken2, a metagenomic classification tool that has previously been used to identify various viral DNA sequences in a range of tissue types [46–48], was developed. The workflow first included read trimming, reads were then filtered using a Kraken2 human genome-specific database, and finally remaining reads were classified using the Kraken2 standard database containing the RefSeq complete genomes for archaea, bacteria, human, viruses, plus UniVec core (a database of vector, adaptor, linker and primer sequences). By applying a minimum cutoff of greater than or equal to two or more reads, thus removing any single match classifications, only four placental metagenomes had reads classified as virus-derived; all classifications were attributed to herpesviruses HHV-6A, HHV-6B and HHV-5 (HCMV) (Fig. 1) and all HHV-classified reads were found in metagenomic samples from full-term births. Alignment to an HHV-6 reference genome showed that the small number of matching reads aligned along the entire HHV-6 genome rather than to one specific region (data not shown).

A second analysis classifying the human-filtered reads against a Kraken2 virus genome-only database identified a significant number of phiX174 reads in each metagenomic sequence (Fig. S1). These were probably introduced as a control in the sequencing protocol. Interestingly, these phiX174 matched reads were not identified in the initial standard database analysis as these reads were classified as bacterial derived phage sequences, and matches were filtered for virus-only classified sequences in that analysis. Reads corresponding to phiX174 were not over-represented in metagenomic samples from full-term births. Therefore, the differences we observed in detection of reads corresponding to human viral genomes (Fig. 1) was unlikely to be related to differences in sequencing of DNA from each placental metagenome.

To ensure that the reads we detected in Fig. 1 were from human herpesvirus genomes, we used Kraken2 to compare reads from placental metagenomes with a database of human herpesvirus genomes that we created for this study (Fig. S2). We found near identical data to those shown in Fig. 1.

To confirm the data produced by the Kraken2 workflow, an alternative method using the GATK PathSeq tool [47] was used. Results showed a very similar classification of the placental metagenomes (Fig. S3). One further low-level match was found to Torque Teno virus, which has previously been found in many human tissue types, although it is not thought to be transmitted across the placenta [49]. Detection of Torque Teno virus reads by GATK, but not Kraken2, may reflect a difference in the content of the databases used by the two tools.

Therefore, using both Kraken2 and GATK workflows, the only reads corresponding to viral DNA genomes that could be confidently identified in placental metagenomes were a small number of reads from the human herpesviruses in samples from full-term pregnancies.

It was interesting to note that reads corresponding to HHV-6A could be detected (SRR1269152, SRR1269157, SRR1269162; Fig. 1). As chromosomal integration of HHV-6A has been reported in pregnant women [22, 26, 28, 32], we hypothesized that the HHV-6A sequences we detected could be from chromosomally integrated virus. We calculated the estimated HHV-6A genome coverage (relative abundance) of the matching reads in SRR1269157 to be 0.36 (Kraken2) or 0.46 (GATK PathSeq), compared to an estimated human genome coverage of 0.79 (assuming the complete read set was human-derived). This possibly suggested that there was one integrated copy of the HHV-6A genome in the SRR1269157 genome. The relative abundance of HHV-6A reads in SRR1269152 was 0.03 or 0.05 (Kranken2 and GATK PathSeq analysis, respectively) and in SRR1269162 was 0.006 for both Kranken2 and GATK PathSeq analysis. However, the number of HHV-6A reads detected in SRR1269152 and SRR1269162 was very low. Thus, it was not possible to draw firm conclusions from these estimations as to whether integrated HHV-6A was or was not present in SRR1269152 and SRR1269162.

### Validation of the metagenomic profiling tool by identifying viral DNA reads in human microbiome metagenomes

Only reads corresponding to the genomes of human herpesviruses could be found in our analysis of placental metagenomes (Figs 1 and S1). To validate the use of Kraken2 in finding viral DNA reads from a range of viral genomes within metagenomic sequencing data, the Kraken2 workflow was applied to human microbiome sequence data that had been deposited in a publicly available database: the Human Microbiome Project [41–43]. In total, 3646 metagenomic samples were analysed. In addition, a study assessing 52 metagenomic profiles from the upper reproductive tract [49] was included.

**Fig. 2.**
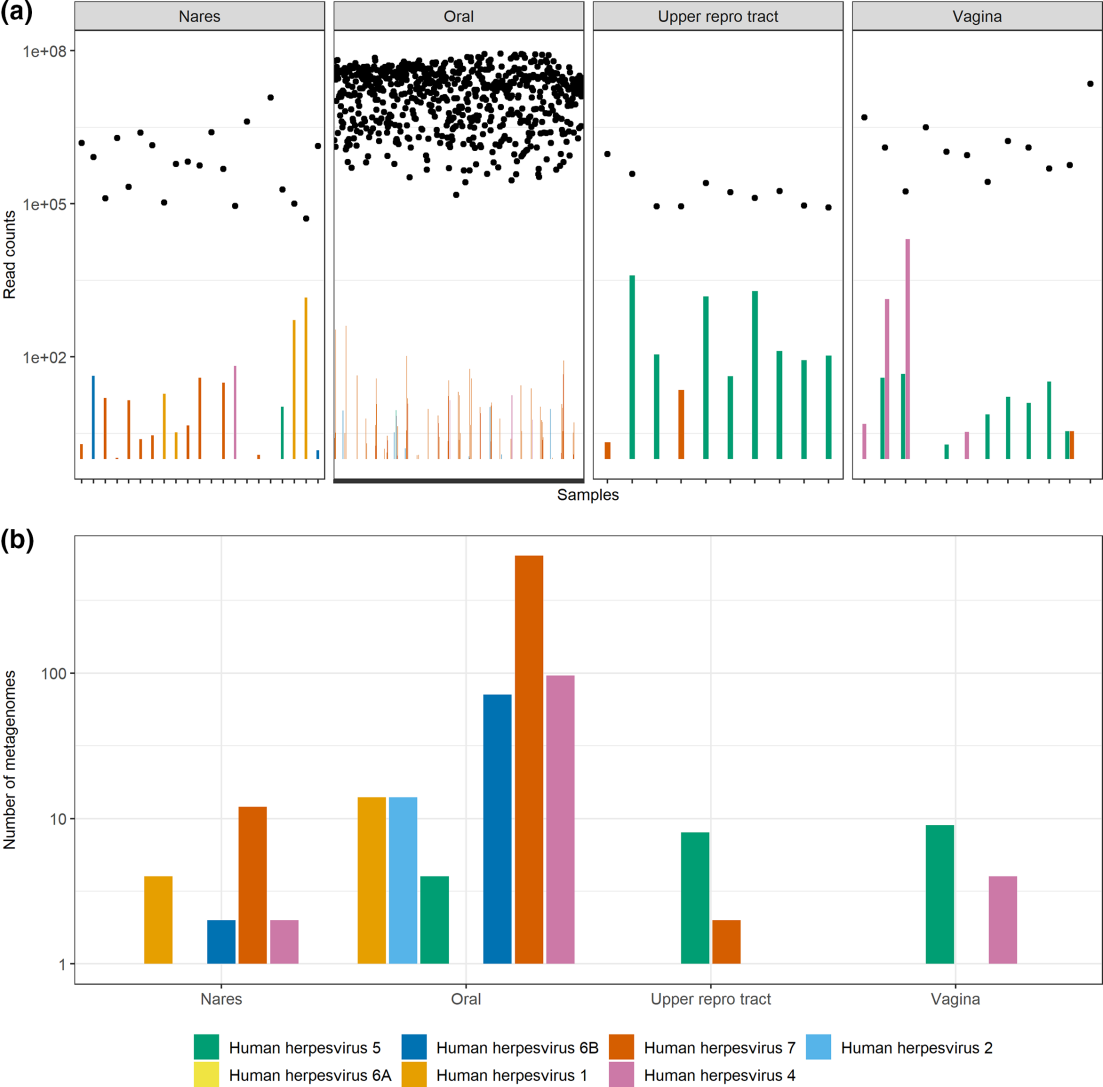
Number of reads corresponding to human herpesviruses in metagenomes from the Human Microbiome Project and a study of the upper reproductive tract. Read counts were divided by sample counts and normalized to 1×10^6^ reads. (**a**) Bars show the classified read counts separated into four general body sites, while the total numbers of reads for each metagenome are plotted with dots above each accession. (**b**) The number of metagenomes where human herpesvirus sequences (two or more reads) are found. In each figure, HSV-1 is shown as human herpesvirus 1, HSV-2 is shown as human herpesvirus 2, EBV is shown as human herpesvirus 4 and HCMV is shown as human herpesvirus 5.

Consistent with a pattern previously observed elsewhere [50], assessment of herpesvirus classifications (Fig. 2) showed a pattern whereby oral samples were largely dominated by HHV-7 sequences and to a lesser extent HHV-4 (EBV) and HHV-6B. HCMV could be found in nares, upper reproductive tract and vaginal samples. Reads corresponding to HHV-1 and HHV-2 (HSV-1 and HSV-2, respectively) genomes were found in nares and oral samples or only oral samples, respectively. Therefore, Kraken2 could detect reads corresponding to genomes of many common human herpesviruses.

**Fig. 3.**
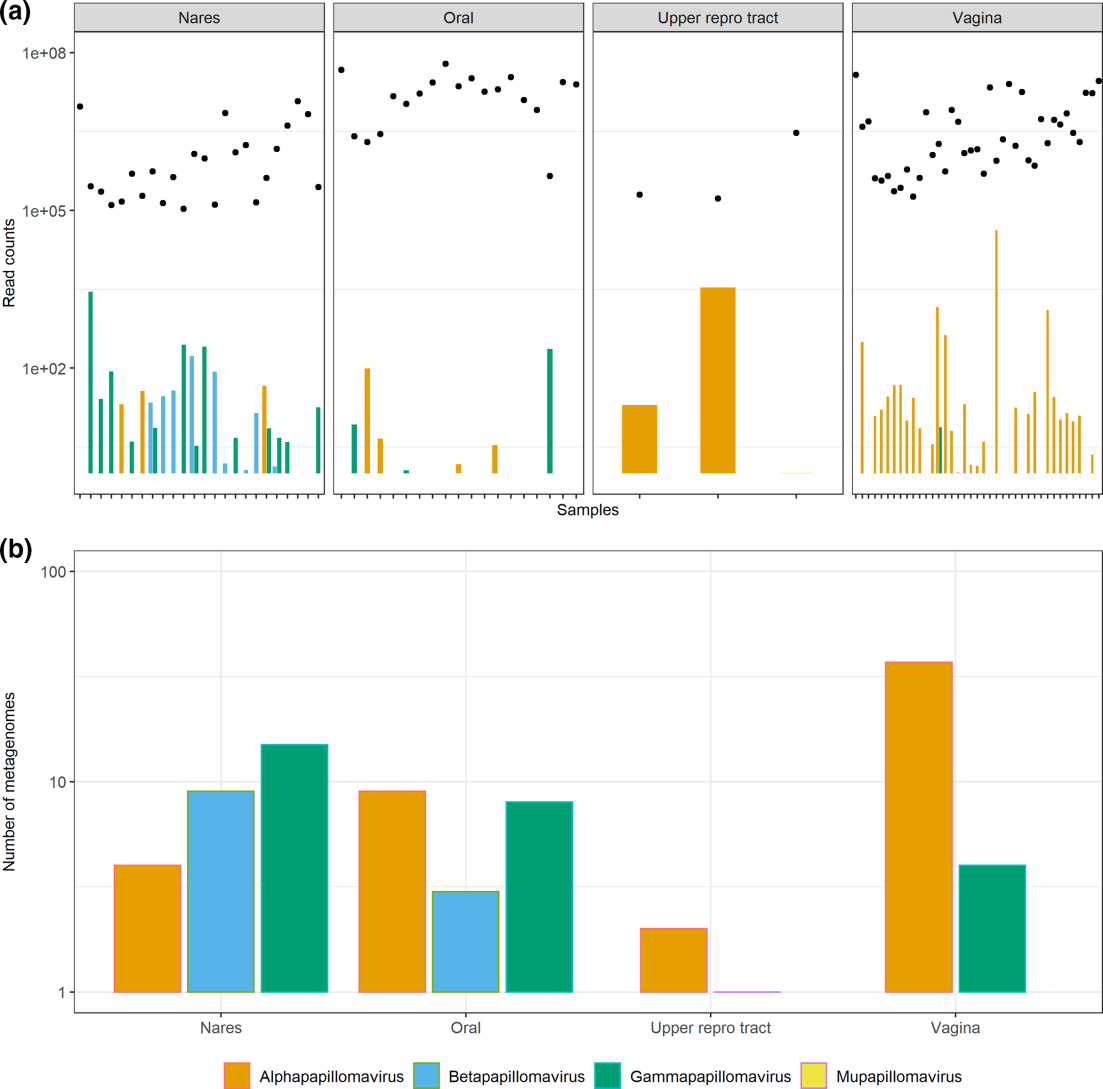
Number of reads corresponding to papillomaviruses in metagenomes from the Human Microbiome Project and a study of the upper reproductive tract. Read counts were divided by sample counts and normalized to 1×10^6^ reads. (**a**) Bars show the classified read counts separated into four general body sites, while the total numbers of reads for each metagenome are plotted with dots above each accession. (**b**) The number of metagenomes where papillomavirus sequences (two or more reads) are found.

To further confirm the validity of the Kraken2 workflow, the distribution of papillomaviruses was assessed in the same metagenome sample set. At the genus level, metagenomes sequenced from the nares were dominated by Betapapillomaviruses and Gammapapillomaviruses (Fig. 3), as has previously been described [51]. Conversely, samples isolated from the upper reproductive tract and vaginal sites were largely dominated by Alphapapillomaviruses.

Therefore, Kraken2 was capable of detecting reads corresponding to genomes from common human DNA viruses such as herpesviruses and papillomaviruses. This suggests that it was likely that Kraken2 could detect reads corresponding to a range of DNA virus genomes if they were present in placental metagenomes.

## Discussion

We sought to understand which human DNA virus genomes could be found in placental tissue and if those DNA genomes differed between patient groups. For example, a difference between metagenomes from placentas of full-term births compared to those from full-term births that had reported antenatal infection during pregnancy. We found that only reads corresponding to the genomes of HHV-6A, HHV-6B and HCMV were identified in four metagenomic samples from full-term births.

The present study was stimulated by recent re-evaluation of the presence of bacteria in placental tissue. The weight of evidence indicates that reports of a unique placental microbiome of commensal bacteria may have been the result of technical issues surrounding preparation and execution of DNA sequencing studies or acquisition of bacteria during labour and/or delivery [16–20, 22]. Thus, it may be possible that the placenta is not routinely colonized by bacteria. As we find very few reads corresponding to human DNA viruses in our analysis, it may be possible that the placenta is not routinely colonized by either bacteria or viruses with DNA genomes. Indeed, our observations are similar to those made elsewhere [26], in so far as only HHV-6A sequences could be found in placental metagenomes.

Our data lead to questions surrounding why herpesviruses known to be vertically transmitted across the placenta (HSV, VZV) were not detected in our experiments and the relevance of detecting HHV-6 and HCMV in our analysis. It is possible that HSV and VZV viral genomes were present in the placental samples, but only at very low numbers that were undetectable at the level of sequencing performed or that these organisms were lost through the DNA extraction protocol. It is equally possible that human herpesvirus infection of the placenta is a rare occurrence and that considerably more placental metagenomes would have to be analysed in order to detect reads corresponding to HSV and VZV genomes. It is interesting to speculate how common herpesvirus infection of the placenta might be. HCMV is perhaps the best studied in this regard. HCMV seroprevalence is high worldwide, with a seroprevalence ranging from 45 to 100 % [52]. However, congenital infection by HCMV occurs in only 0.2–2 % of live births [52]. Therefore, HCMV infection of the placenta may not be common, in line with observations made in our study and elsewhere [26].

It is interesting to contrast our observations with a previous report that HCMV and HSV genomes could be routinely observed in placental and decidual tissue obtained from both first trimester terminations of healthy pregnancies and in placental tissue from second trimester terminations of healthy pregnancies (HCMV in 28 and 26 % of samples, respectively; HSV-1 in 3 and 3 % of samples, respectively; HSV-2 in 6 and 13 % of samples, respectively) [25]. It is possible that the differences in our observations are due to experimental differences that relate to the sensitivities of the assays used (PCR vs. metagenomic profiling) or the tissue that was examined (in the aforementioned work [25], several tissue samples were taken from random sites in the placental and decidual tissue, whereas the DNA analysed here was taken from the same region of each placenta). Furthermore, the DNA analysed here was from near full-term or full-term spontaneous births, whereas the tissue analysed in the aforementioned study [25] was from first and second trimester terminations of healthy pregnancies. This implies that DNA virus infection of the placenta may be more common early in pregnancy, during the first and second trimesters, or that there are areas of the placenta enriched in virus-infected cells.

We found reads corresponding to HHV-6 genomes in four of the placental metagenomic samples analysed. We considered if the presence of reads corresponding to the HHV-6 genomes was due to contamination of the placental metagenomes we had analysed, but know of no plausible route that would allow HHV-6 DNA to contaminate the placental metagenomic data we analysed. We know of no environmental source that would contain HHV-6 viruses. It is possible that HHV-6 viruses could have been introduced during preparation of DNA from placental tissue. HHV-6A can cause dermal infections, but given the precautions taken during metagenomic sample processing [5], it is unlikely that dermal shedding of HHV-6A into placental tissues is a route of contamination in this case. HHV-6A is not known to be transmitted in the air. HHV-6A is not known to be a contaminant found in DNA preparation kits, which was a probable source of bacterial contamination of placental tissue in studies elsewhere [16]. Therefore, we suggest that the presence of reads corresponding to HHV-6 genomes in our analysis is unlikely to be due to contamination of the metagenomic sequences that we have analysed. Furthermore, DNA sequences corresponding to HHV-6 genomes have been reported in placental genomes prepared using stringent isolation procedures [22, 26].

Our observation that reads corresponding to HHV-6 were found in placental metagenomes suggests that these viruses can potentially replicate in the placenta. Several points support HHV-6A infection of the placenta. The aforementioned virus is found in the betaherpesvirus sub-family of the herpes viruses and, as such, is related to the vertically transmitted TORCH pathogen HCMV [53]. It has been demonstrated that HHV-6A can replicate within placental tissue *ex vivo* [33], HHV-6 DNA can be found in foetal tissue, umbilical cord blood and villous tissue [30], and widespread infection of pregnant women with HHV-6A has been reported, which may be associated with disease [26–33, 54]. Importantly, as HHV-6A can integrate into chromosomal DNA, it is possible that the presence of reads corresponding to HHV-6A genomes that we detect in placental metagenomes was not due to infection of the placenta, but from HHV-6A genomes integrated into the genomes of pregnant women, which has been reported elsewhere [22, 26, 28, 32].

A relationship between chromosomal integration of HHV-6A in placenta and pre-eclampsia has been established [26]. We did not detect HHV-6A genomes in placental samples from either pre-term births or pregnancies with antenatal infection, suggesting no obvious link between HHV-6A infection and those patient groups. However, a larger study may be required to confirm this.

Another question we sought to answer was what breadth of DNA viruses might be found in placental tissue. As outlined above, viruses from a number of DNA virus families can replicate in cells derived from placental tissue *in vitro* [34, 36, 38, 39], although neither Adenovirus or Papillomavirus DNA has been detected in a range of pregnancies [26]. In our study, only reads corresponding to the HHV-6A/6B and HCMV genomes were found in placental metagenomes. Alternatively, it is possible that a number of other viral DNA genomes are in the placental metagenomes we analysed, but present at levels that were undetectable at the level of sequencing performed. It is also possible that placental infection with DNA viruses other than herpesviruses is rare, and further study of a range of placental tissue may reveal the presence of these viruses. Importantly, our data may indicate that replication of some DNA viruses in placental cells *in vitro* may not reflect the ability of those viruses to infect placental tissue *in vivo*.

Furthermore, we wished to examine the potential relationship between the presence of non-commensal bacterial infection and virus infection of the placenta. We found no obvious relationship between the presence of any virus and antenatal infection during pregnancy. Similarly, we found no obvious relationship between virus infection and pre-term birth. It is possible that these links exist, but were not observed here due to the caveats discussed in the preceding paragraphs, for example study size and levels of detection. That said, our data may imply that there is no obvious link between pre-term birth or antenatal infection and DNA virus infection.

Further study of virus infection of the placenta is warranted. As discussed here, to detect herpesviruses and other DNA viruses, it is likely that investigation will have to be expanded to examine much larger numbers of tissue. Furthermore, based on the points we discuss here, consideration should be given to examining tissue from both early trimesters of pregnancy and tissue from full-term pregnancy. Finally, there are several important vertically transmitted viruses that have RNA genomes (e.g. rubella virus and Zika virus). The methodology used here would not detect these RNA genomes. To fully understand virus infection of the placenta it will be necessary in future studies to consider viruses with both DNA and RNA genomes.

## Supplementary Data

Supplementary material 1

Supplementary material 2
