# Supplementary material for: Metagenomic profiling of placental tissue suggests DNA virus infection of the placenta is rare
Source: J Gen Virol. 2021 Nov 1;102(11):001677. doi: 10.1099/jgv.0.001677 (PMC8742990; doi:10.1099/jgv.0.001677)

Supplementary Data

Metagenomic profiling of placental tissue suggests DNA virus infection of the placenta is rare

Adam A Witney, Sean Aller and Blair L Strang

Supplementary Figure 1 Number of reads corresponding to phiX174 in the placental metagenomes. The number of reads corresponding to phiX174 in each placental metagenomic sample was determined using Kraken2. The sample accession of each metagenomic sample was noted on each x-axis. Metagenomic samples came from either full-term pregnancies, pre-term pregnancies, full term pregnancies that had reported antenatal infection or pre-term pregnancies that had reported antenatal infections (Pt + inf).

Supplementary Figure 2 Number of reads corresponding to human herpesvirus genomes in placental metagenomes using a human herpesvirus database. The number of reads corresponding to human herpesvirus genomes in each placental metagenomic sample was determined by comparing reads to a database of human herpesvirus genomes using Kraken2. The sample accession of each metagenomic sample was noted on the x-axis. Metagenomic samples came from either full-term pregnancies, pre-term pregnancies, full term pregnancies that had reported antenatal

infection or pre-term pregnancies that had reported antenatal infections (Pt + inf). HCMV is shown as human herpesvirus 5.

Supplementary Figure 3 Number of reads corresponding to virus genomes in placental metagenomes using the GATK PathSeq tool. The number of reads corresponding to human viruses in each placental metagenomic sample was determined using GATK PathSeq. The sample accession of each metagenomic sample was noted on the x-axis. Metagenomic samples came from either full-term pregnancies, pre-term pregnancies, full term pregnancies that had reported antenatal infection or pre-term pregnancies that had reported antenatal infections (Pt + inf). HCMV is shown as human betaherpesvirus 5.

Table S1 Accession numbers for Human Microbiome Project data. See excel sheet the accompanies this manuscript.

Supplementary figure 1

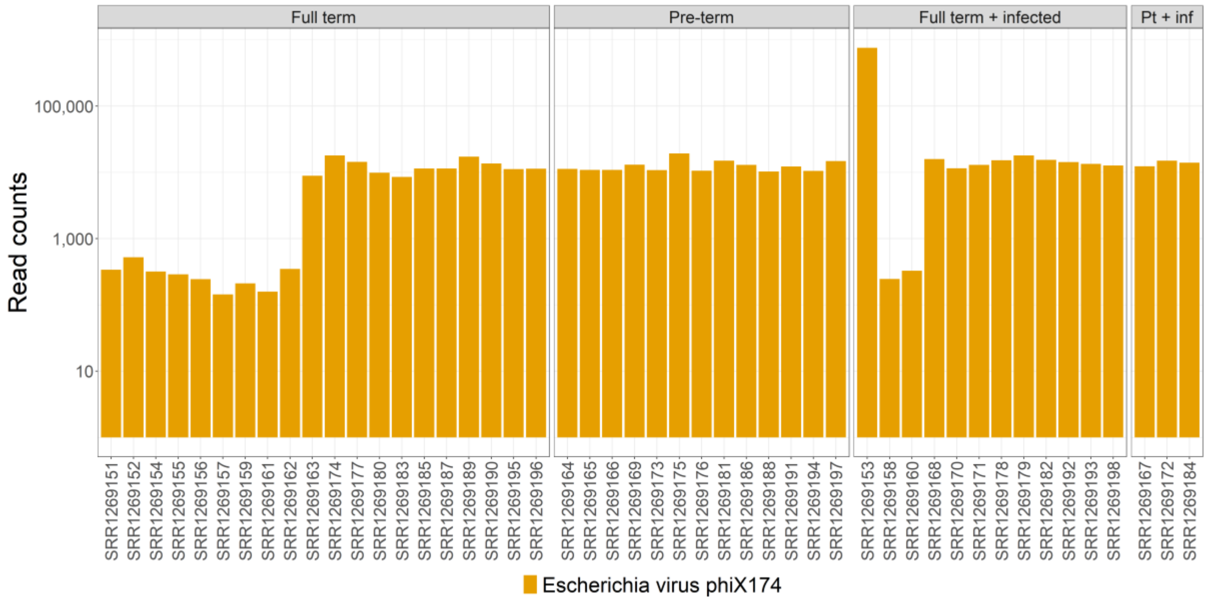

Supplementary figure 2

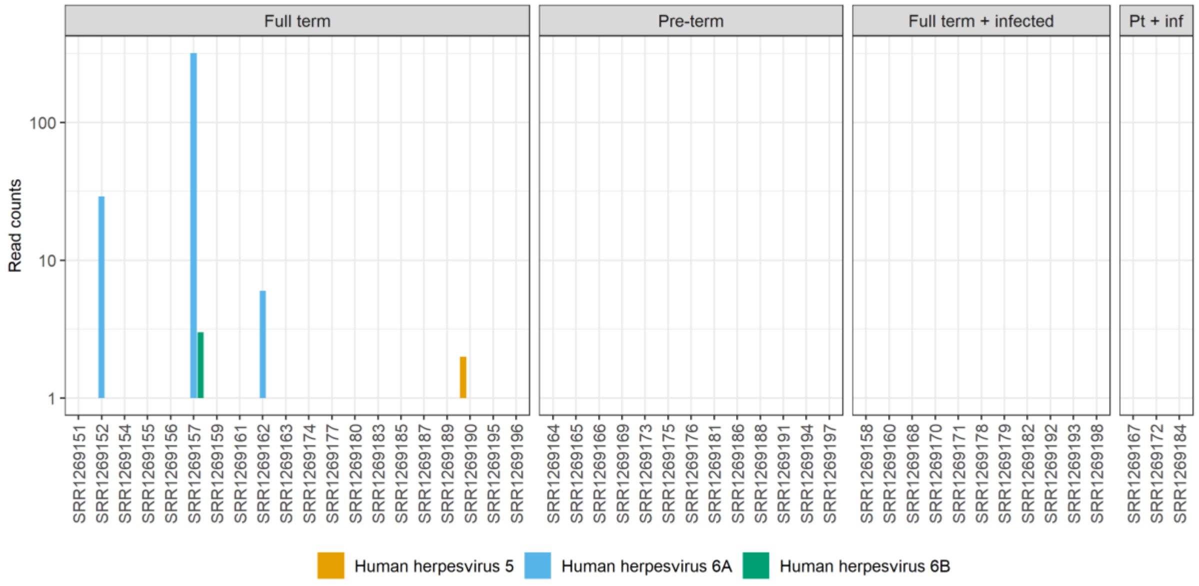

Supplementary figure 3

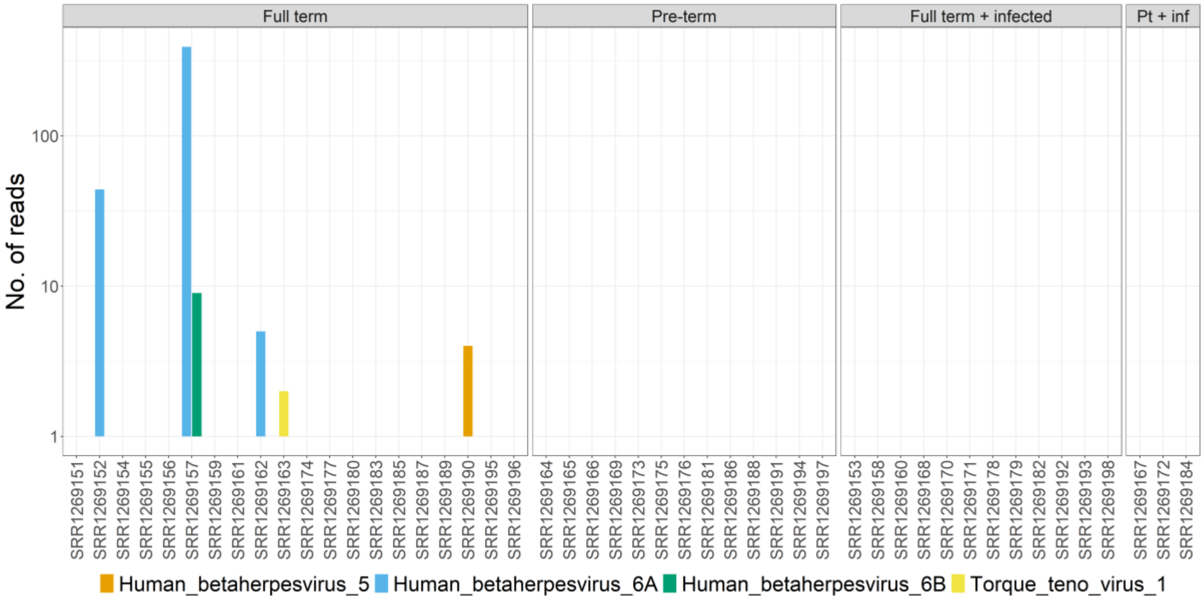

Supplement: Supplementary material 1 [file jgv-102-1677-s001.pdf]
